# Valproic acid exerts specific cellular and molecular anti-inflammatory effects in post-operative conjunctiva

**DOI:** 10.1007/s00109-018-1722-x

**Published:** 2018-11-19

**Authors:** Li-Fong Seet, Li Zhen Toh, Sharon N. Finger, Stephanie W. L. Chu, Tina T. Wong

**Affiliations:** 1 Ocular Therapeutics and Drug Delivery, Singapore Eye Research Institute, The Academia, 20 College Road, Singapore, 169856 Singapore; 2 Department of Ophthalmology, Yong Loo Lin Sc hool of Medicine, National University of Singapore, Singapore, Singapore; 3 Duke-NUS Medical School Singapore, Singapore, Singapore; 4 Glaucoma Service, Singapore National Eye Center, 11 Third Hospital Avenue, Singapore, 168751 Singapore; 5 School of Materials Science and Engineering, Nanyang Technological University, Singapore, Singapore

**Keywords:** Conjunctiva, Glaucoma filtration surgery, Histone deacetylase inhibitor, Inflammation, NF-kB, TNF-α, Valproic acid

## Abstract

**Abstract:**

Valproic acid (VPA) is a histone deacetylase inhibitor used clinically for neurological disorders. It is also potentially useful as anti-fibrotic therapy as it reduced collagen deposition in the post-operative conjunctiva. In this study, we further evaluated the effects of VPA on post-operative inflammation using the mouse model of conjunctival scarring. VPA, injected into the subconjunctiva immediately after surgery, did not cause any adverse tissue response when examined by live microscopy and produced an apparent reduction of proinflammatory and proangiogenic markers in immunohistological examinations. In-depth analyses of the treated operated tissues revealed that VPA selectively inhibited the CD45^high^F4/80^low^ macrophage subset as well as the production of specific proinflammatory cytokines/ chemokines, including CXCL1, IL-5, IL-6, and IL-10 which were reduced by ≥ 2.0-fold. VPA also specifically reduced tissue NF-кB2 p100 protein by mean 3.87-fold. On conjunctival fibroblasts, VPA treatment resulted in decreased secretion of specific cytokines, including CCL2, VEGF-A, and IL-15. In the presence of TNF-α, VPA inhibited the induction of specific cytokines/chemokines, notably CCL5 and VEGF-A, as well as NF-кB2 p100. In corroboration, VPA suppressed TNF-α stimulation of NF-кB reporter transcription by 1.51-fold. These data indicate that VPA can modulate both proinflammatory cellular and molecular targets in a selective manner and may therefore attenuate surgery-induced conjunctival inflammation. These and previous findings suggest that, by suppressing key mediators of both inflammation and fibrosis, VPA is a useful therapeutic for improving surgical outcome involving the conjunctiva.

**Key messages:**

- VPA inhibited recruitment of a CD45^high^F4/80^low^ macrophage subset.
- VPA reduced chemokine and cytokine levels in treated tissues.
- VPA selectively suppressed tissue NF-кB2 p100 levels.
- VPA suppressed TNF-α induction of chemokines, cytokines and NF-кB2 p100 expression.
- VPA suppressed TNF-α stimulation of NF-кB reporter.

## Introduction

Glaucoma filtration surgery (GFS), performed to relieve elevated intraocular pressure, commonly fails due to scarring at the sclerostomy site, involving the conjunctiva [1]. Excessive or persistent inflammation after GFS is associated with high risk of scarring. Steroids as well as other anti-inflammatory drugs, applied systemically, topically, or in the subconjunctiva, have been suggested as post-operative management to prevent failure [2–4]. However, these regimens commonly involve taking these immunosuppressive/anti-inflammatory drugs for prolonged periods and steroids, in particular, are associated with potentially serious adverse effects [5]. Hence, alternative anti-inflammatory agents, with better safety profiles, and ideally, coupled with anti-fibrotic properties, would be advantageous for reducing the risk of scarring and improving the clinical outcome in GFS.

In this study, we determined the efficacy of a well-known histone deacetylase (HDAC) inhibitor, valproic acid (VPA), in modulating inflammation in the post-operative conjunctiva. HDAC inhibitors have been widely investigated as anti-inflammatory agents [6]. Since these inhibitors regulate both histone and non-histone acetylation, and specific cell types respond to unique proinflammatory stimulus with the induction of distinct gene panels that are regulable by selective HDACs, varied effects of HDAC inhibitors on inflammation were frequently reported. The efficacy of individual HDAC inhibitor for a specific inflammatory condition must therefore be addressed in its unique biological or disease context. For VPA, its multifarious anti-inflammatory effects have been reported on animal models of diverse inflammatory diseases [7–11].

The safety profile of VPA is well documented as it has been used for over four decades for the treatment of neuropsychiatric disorders [12]. VPA is also potentially useful as an anti-fibrotic, as we have previously found that VPA ameliorated fibrosis by suppressing collagen production [13] in the mouse model of GFS/ conjunctival scarring [14]. The post-operative response of this experimental surgery model closely mimicked that of patients with GFS [15] and consists of an early inflammatory phase that precedes repair or fibrosis [16]. At the same dosage previously shown to be effective for inhibiting collagen deposition [13], we demonstrate in this study that subconjunctival injection of VPA modulated tissue macrophage composition and chemokine/cytokine levels, as well as repressed specific NF-kB expression. In conjunctival fibroblasts, which we have shown previously to faithfully replicate in vivo responses when exposed to proinflammatory or profibrotic stimulus [16], VPA not only reduced specific cytokine production in uninduced cells but also has the capacity to suppress TNF-α induction of multiple cytokines. These data therefore suggest that VPA may be useful for mitigating inflammation following conjunctival surgery and implies potential benefits in pre-, intra-, and post-operative application. Given its capacity to ameliorate both processes of inflammation and scarring in GFS, VPA is a potential replacement for conventional anti-inflammatory cum anti-fibrotic therapies for GFS.

## Materials and methods

### Mouse model of conjunctival fibrosis

All experiments with animals were approved by the Institutional Animal Care and Use Committee (IACUC) and treated in accordance with the Association for Research in Vision and Ophthalmology (ARVO) Statement on the Use of Animals in Ophthalmic and Vision Research. NIH3T3/BL6 mice, originally obtained from the National University of Singapore Centre for Animal Resources and subsequently bred in the SingHealth Experimental Medicine Centre (Singapore), were used. Mice used include both males and females, with age ranging from 8 to 10 weeks old. Experimental surgery was performed as described previously [14]. Valproic acid from Sigma-Aldrich Co. (St. Louis, MO), dissolved in PBS, was injected at 300 μg/ml in 5 μl volume into the conjunctiva immediately after surgery. PBS was similarly injected as control. Fucithalmic ointment (Leo Pharmaceutical Products, Ballerup, Denmark) was instilled at the end of the procedure to prevent infection. On day 2 post-surgery, mice were euthanized by intraperitoneal injection of pentobarbitol sodium, at 100 mg/kg body weight, before conjunctival tissues were harvested.

### Live imaging of mouse eyes

Mice were anesthetized and imaged on day 2 post-surgery. Imaging was performed using the slit lamp (Righton LED slit lamp MW50D, Right Mfg Co Ltd., Japan), anterior segment-optical coherence tomography (AS-OCT) (Optovue RTVue100-2 Fourier domain optical coherence tomography system, Optovue Inc., Fremont, CA, USA) and the confocal microscope (HRT3 microscope, Heidelberg Engineering, Heidelberg, Germany).

### Histology and immunofluorescence analyses of cryosections

Each enucleated eye ball was fixed with paraformaldehyde and then placed in a slurry of optimal cutting temperature (OCT) compound in cryomold before freezing in dry ice and storage in a − 80 °C freezer until ready for sectioning using the Microm HM550 (Carl Zeiss Ltd). Five-micrometer cryosections of day 2 post-operated eye tissues stained with hematoxylin and eosin (H&E) staining was visualized as described previously [14]. A total of 3 eyes for each condition were evaluated. Acetyl-histone H3, CD45 and F4/80 antibodies were obtained from Merck Millipore (Darmstadt, Germany), BD Pharmingen (San Diego, CA, USA) and Abcam Plc (Cambridge, UK), respectively. Isolectin B4-Alexa Fluor 568 conjugate was from Molecular Probes Inc. (Eugene, OR, USA). Labeling by the primary antibodies was detected using secondary antibodies conjugated to Alexa Fluor-594 (red fluorescence) or Alexa Fluor-488 (green fluorescence), both obtained from Invitrogen Corp. (Thermo Fisher Scientific Inc., Waltham, MA, USA). Nuclei were visualized by mounting the cells in DAPI-containing Vectashield mounting medium (Vector Laboratories, CA, USA). Labeled cells were visualized using the Zeiss Imager.Z1 microscope (Carl Zeiss Inc., USA).

### Flow cytometry

Experimental surgery was performed with each set of experiment consisting of a pool of 20 operated eyes injected with PBS and a corresponding pool injected with VPA. A total of 5 experimental sets was performed (*n* = 5 per treatment; both eyes of each mouse were operated and treated similarly; total 100 mice were used). Harvesting and processing of operated conjunctival tissues, as well as immunolabeling and analyses of cells were performed as described previously [16]. CD45 antibody conjugated to allophycocyanin (APC) (clone 30-F11), F4/80 antibody conjugated to phycoerythrin (PE) (clone BM8), and CD11b antibody conjugated to eFluor450 (clone M1/70) were all obtained from eBioscience Inc. (San Diego, CA, USA). Isotype controls for gating were rat IgG conjugated to APC (BD Pharmingen), PE (BD Pharmingen), or eFluor450 (eBioscience). Staining with 7-AAD (ViaProbe; BD Biosciences) was used to exclude non-viable cells with live cells being defined as 7-AAD-negative. Immunolabeled cells were acquired on the BD FACSVerse flow cytometer (BD Biosciences) and analyzed using FlowJo 7.6.

### Primary conjunctival fibroblast cell culture and treatments

Primary conjunctival fibroblasts, obtained from the eyes of C57BL6/J mice, were cultured as described previously [14], with one exception: 5% fetal bovine serum was used in cultures designated for multiplex cytokine assays to reduce background signals. Treatment with VPA (Sigma-Aldrich Co., St. Louis, MO) was carried out at 300 μg/ml (or 2 mM) for 48 h unless otherwise indicated. Stimulation with TNF-α was performed by treating the cells with 20 ng/ml TNF-α (R&D Systems, Minneapolis, MN, USA) for 48 h. Co-treatment with VPA and TNF-α was performed using the same indicated concentrations.

### Multiplex cytokine assay

For tissue analyses, experimental surgery was performed to obtain a total of five sets of data for each condition (PBS or VPA treatment), with each set consisting of a pool of five operated eyes from five animals (*n* = 5 per treatment; only the left eye of each mouse was operated on and treated; total 50 mice were used). Tissues were harvested and processed as described previously [16]. For in vitro analyses, fibroblast culture supernatants were first concentrated about 5-fold using Vivaspin concentrators (Vivaproducts, Inc., Littleton, MA, USA). The pre-mixed 32-plex, Milliplex MAP mouse cytokine/chemokine antibody array (Merck Millipore, Billerica, MA) was incubated with the tissue lysates or culture supernatants according to instructions by the manufacturers. Cytokine levels measured using the Bio-Plex 200 system (Bio-Rad Laboratories, Hercules, CA) were then normalized to total protein content of each lysate or culture supernatant determined using the Bradford protein assay (Bio-Rad Laboratories, Inc., Hercules, CA, USA).

### Immunoblotting

For in vivo analyses, three groups of tissues for each treatment condition, each group consisting of pooled tissues from five eyes of five independent animals, were analyzed (*n* = 3 per treatment; total 30 mice were used). For in vitro analyses, three independent sets of experiments using different batches of primary cells, with each set comprising of the four treatment conditions (control, TNF-α only, VPA only, and TNF-α + VPA), were performed (*n* = 3). Processing of operated tissues or cultured fibroblasts and analyses of lysates by immunoblotting were performed as previously described [16]. For tissue protein analyses, NF-кB1 p105 (#4717) and NF-кB2 p100 (sc-7386) antibodies were from Cell Signaling Technology (Danvers, MA) and Santa Cruz Biotechnology, Inc. (Santa Cruz, CA), respectively. For fibroblast protein analyses, NF-кB1 p105 (sc-8414), phospho-NF-кB1 p105 (Ser933) (#4806), NF-кB2 p100 (ab31432), and phospho-NF-кB2 p100 (Ser865) (ab31474) antibodies were from Santa Cruz Biotechnology, Cell Signaling Technology, and the latter two from Abcam Plc, respectively. RelA (C-20; sc-372), RelB (C-19; sc-226), and GAPDH (sc-25778) antibodies were all obtained from Santa Cruz Biotechnology. c-Rel antibody (AF2699) was obtained from Abcam Plc. Horseradish peroxidase (HRP)-conjugated secondary antibodies were from Jackson Immunoresearch Laboratories, Inc. (West Grove, PA). Densitometric analyses were performed and values were normalized using the housekeeping GAPDH.

### Reporter gene assays

The NF-қB cignal reporter assay kit was purchased from Qiagen (Valencia, CA). Plasmids were transfected into conjunctival fibroblasts using the P2 primary cell 4D Nucleofector kit L (Lonza, Basel, Switzerland) and the 4D-Nucleofactor X unit (Lonza) according to manufacturer’s protocol. Transfected cells were allowed to recover and treated the next day with PBS, VPA, and/or TNF-α for 24 h. Luciferase activity was then measured via the Tecan Infinite M200 reader (Tecan Trading AG, Switzerland) using the Dual-Glo® Luciferase Assay system (Promega) according to the manufacturer’s protocol. The values were normalized to Renilla luciferase activity provided by expression from the internal control included in the NF-қB assay kit. Three independent assays using independent batches of cells were performed. Data is presented as the mean of the fold changes derived from the three sets of independent experiments for each condition.

### Statistical analysis

All data are expressed as mean ± standard deviation (SD) of *n* = 5 independent tissue samples, or *n* = 3 independent sets of cell culture experiments, the latter performed in triplicates for each set. Where only two treatment conditions were compared, the significance of differences between the two conditions was determined by the two-tailed Student’s *t* test using the Microsoft Excel 5.0 software. Where more than two treatment conditions were compared, the significance of differences between the conditions was determined by one-way ANOVA using SPSS statistics. Bonferroni post hoc adjustment was applied to determine which conditions were significantly different from each other. Statistical significance was defined as *p* < 0.05.

## Results

### VPA treatment did not stimulate a tissue response in the conjunctiva

**Fig. 1:**
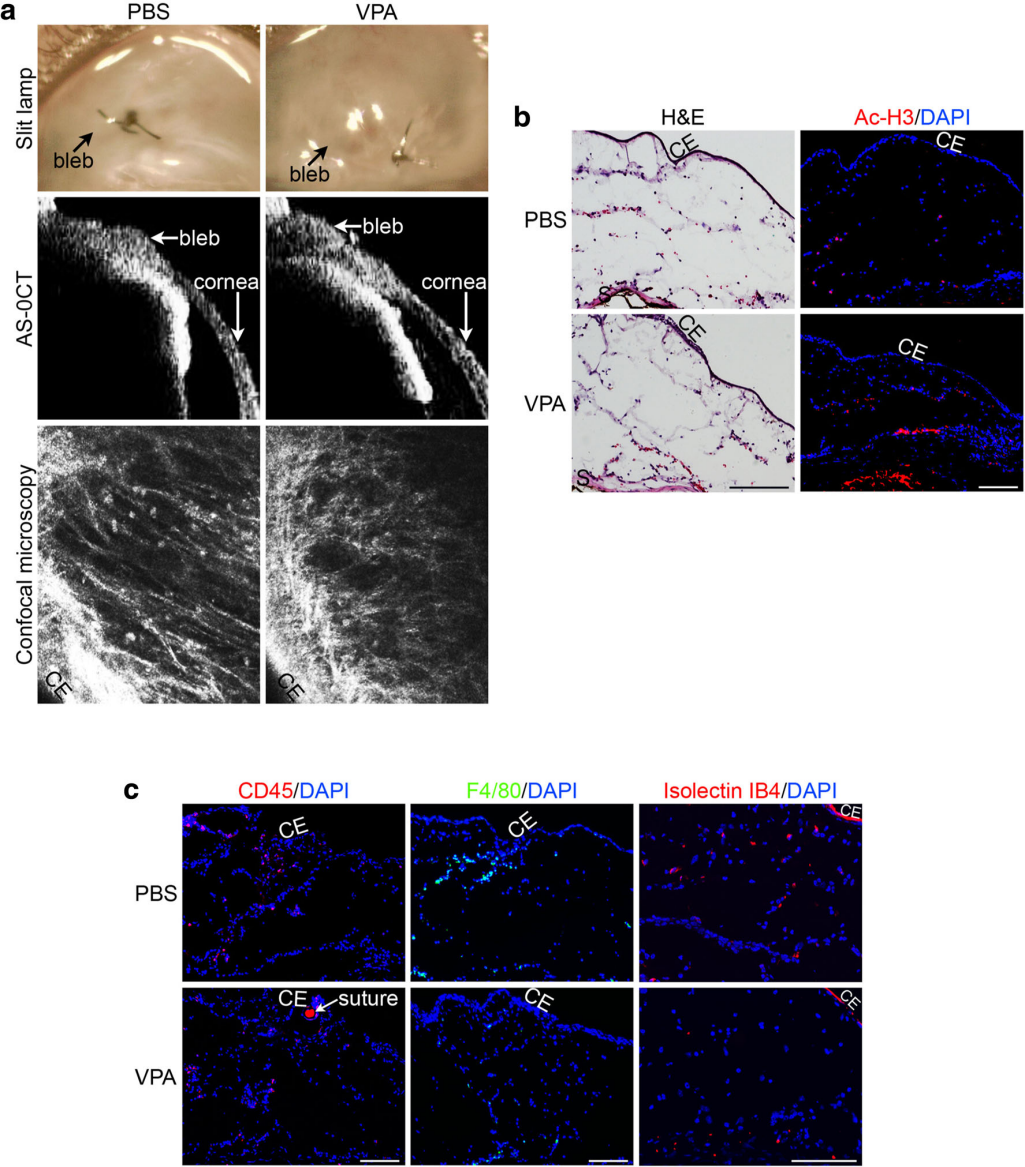
Morphology and histology of the operated conjunctiva treated with VPA. **a** Imaging of live operated mouse eyes treated with PBS (control) or VPA. The same operated eye was imaged by slit lamp, AS-OCT, and confocal microscopy on day 2 post-surgery. Operated conjunctiva can be visualized as blebs. **b** Immunohistological evaluation of day 2 operated conjunctiva treated with PBS (control) or VPA. Visualization of histological structures and histone acetylation status in the operated conjunctival cryosections by staining with hematoxylin and eosin (H&E) and immunofluorescence staining with the acetyl-histone H3 antibody (Ac-H3, red) respectively. **c** Visualization of inflammatory/endothelial cells in the operated conjunctival cryosections by immunofluorescence staining with CD45 (red) and F4/80 (green) antibodies, as well as staining with isolection IB4 fluorescently labeled conjugate (red). Nuclei were visualized by DAPI staining (blue). CE conjunctival epithelium. Scale bar = 100 μm

The day 2 VPA-treated post-operative mouse conjunctiva, visible as a filtering bleb under the slit lamp, did not demonstrate increased vascularity or adverse tissue response compared with control PBS-treated bleb (Fig. 1a, top panel). Anterior segment-optical coherence tomography (AS-OCT) imaging of the bleb also revealed no apparent tissue reaction to VPA (Fig. 1a, middle panel). Scanning of the treated blebs by live confocal microscopy revealed the presence of hyperreflective dots that may represent inflammatory cells in both PBS- and VPA-treated blebs, with no conspicuous difference between the two (Fig. 1a, bottom panel).

Evaluation of frozen bleb cryosections revealed an increase in the acetylated forms of histone H3 as evidence of inhibition of HDAC activity by VPA (Fig. 1b, right panel). Inflammatory cells in the PBS- and VPA-treated were visualized by immunolabeling for CD45-positive proinflammatory cells (Fig. 1c, left panel) or F4/80-positive macrophages (Fig. 1c, middle panel). Proinflammatory cells appear to congregate in focal areas of the operated bleb and VPA treatment seemed to produce fewer of these clusters. Moreover, there appeared to be fewer isolectin IB_4_-binding cells in the VPA-treated blebs (Fig. 1c, right panel), suggesting that there are potentially less perivascular and endothelial cells, and hence the possibility that VPA may suppress angiogenesis in the post-operative bleb.

### VPA reduced the CD45^high^F4/80^low^ macrophage subset in treated operated conjunctiva

**Fig. 2:**
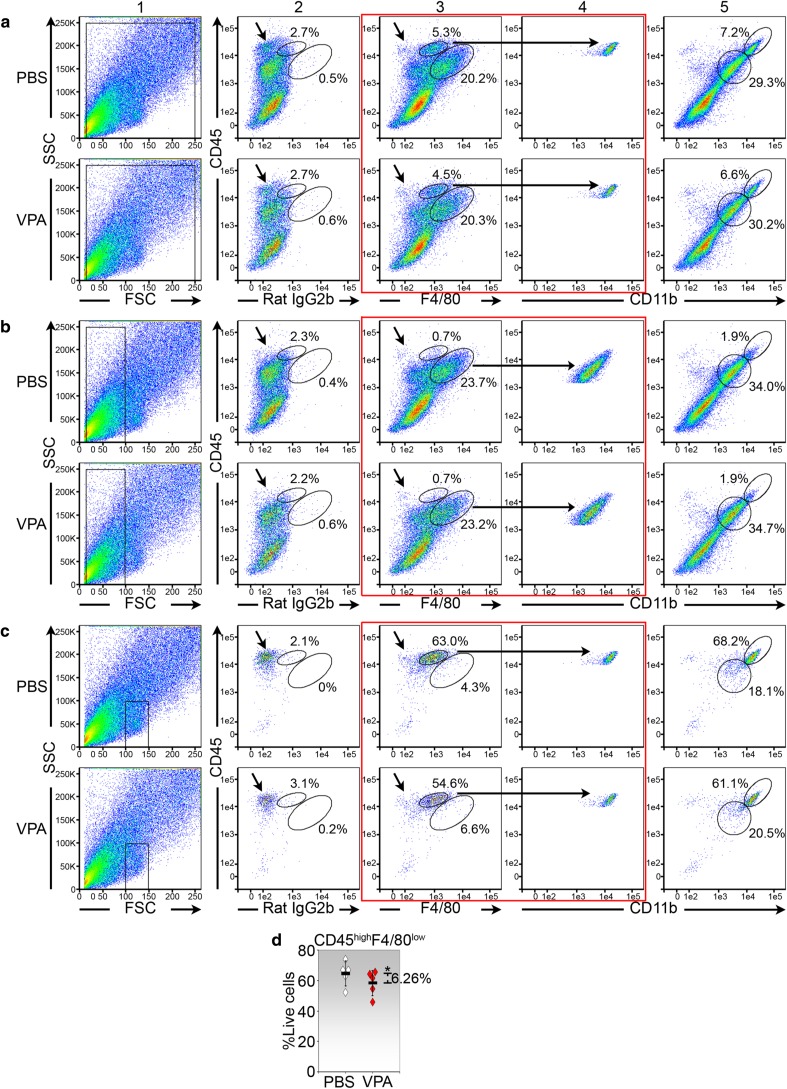
Alteration in specific macrophage subset in the operated conjunctiva treated with VPA. Cells isolated from day 2 operated tissues, treated with either PBS (control) or VPA, were first gated as shown in panel 1 based on SSC vs FSC, followed by gating for live cells using 7-AAD (not shown). Panel 2 shows scatter from live cells stained with negative control IgG2b antibodies. Panel 3 shows positive staining for both CD45 and F4/80 antibodies, with gates representing CD45^high^F4/80^low^ (smaller oval) or CD45^low^F4/80^high^ (larger oval). Panel 4 shows scatter derived from the gate indicated by the long horizontal arrow in panel 3. Panel 5 shows scatter from live cells that label positive for both CD45 and CD11b antibodies. **a** Gating for all cells to reveal CD45^high^F4/80^low^ or CD45^low^F4/80^high^ subsets. **b** Gating for smaller FSC cells to reveal mainly the CD45^low^F4/80^high^ subset. **c** Gating for larger FSC cells to reveal mainly the CD45^high^F4/80^low^ subset. **d** Five independent sets of experiments, with each set of experiment consisting of either PBS or VPA treatment, were analyzed for the CD45^high^F4/80^low^ subset based on the gating shown in **c**. The fold difference in the % live CD45^high^F4/80^low^ subset comparing VPA and PBS treatment is indicated. *p < 0.05

Since macrophages are key cellular mediators of inflammation, we further examined the effect of VPA on these cells by flow cytometry. First of all, cells that were positively immunolabeled for CD45, a marker for cells of hematopoietic origin, were not significantly different between PBS and VPA treatments (data not shown). To specifically identify macrophages, we co-labeled the tissue cells with F4/80 antibody, a commonly used murine macrophage marker [17, 18]. We noticed that operated conjunctival tissues, irrespective of PBS or VPA treatment, contained two distinguishable populations of CD45^+^F4/80^+^ cells: a major subset that expressed lower CD45 and higher F4/80, CD45^low^F4/80^high^ (Fig. 2a, panels 2 and 3, large oval), and an approximately 4- to 5-fold smaller subset that expressed higher CD45 and lower F4/80, CD45^high^F4/80^low^ (Fig. 2a, panels 2 and 3, small oval). Two subsets of CD45^+^CD11b^+^ cells, CD45^high^CD11b^high^ and CD45^low^ CD11b^low^ also exist in the operated conjunctiva (Fig. 2a, panel 5). Notably, the CD45^high^F4/80^low^ subset was also CD11b^high^, a marker for activated macrophages [19] (Fig. 2a, panel 4). A cursory evaluation suggested that CD45^high^F4/80^low^ cells may be fewer in the VPA-treated blebs while the major population of CD45^low^F4/80^high^ cells was similar to PBS-treated controls (Fig. 2a, panel 3).

Since global, non-selective, side scatter versus forward scatter (SSC vs FSC) gating resulted in a high level of background staining associated with the CD45^high^F4/80^low^ population, we proceeded to determine the major SSC vs FSC location of each CD45^+^F4/80^+^ subset. Majority of the CD45^low^F4/80^high^ population resided in the lower FSC region where CD45^high^F4/80^low^ cells were minimally present (Fig. 2b). These CD45^low^F4/80^high^ cells, which were also CD11b^low^ (Fig. 2b, panel 4), were not significantly different between VPA and PBS treatments (data not shown).

We ascertained that CD45^high^F4/80^low^ cells were larger cells that were not detected in the lower FSC region but were principally located in the region characterized by higher FSC, which incidentally, appeared to contain a discrete population of cells (Fig. 2c, panel 1). Gating as such produced the lowest non-specific staining for the CD45^high^F4/80^low^ cells (Fig. 2c, panels 2 and 3). F4/80 was decidedly a better marker than CD11b to define the macrophage subset in this FSC-SSC region since a relatively large proportion of CD45^low^CD11b^low^ was also located here (approximately 20%) (Fig. 2C, panel 5). When gated this way, we found that VPA significantly reduced the numbers of the CD45^high^F4/80^low^ subset by 3–9% in five independent interrogations, or a mean of 6.26% of live cells present in the selected FSC-SSC region (Fig. 2d). Taken together, these data suggest that VPA alters the proinflammatory cell composition of the operated conjunctiva in a selective manner by inhibiting a more highly activated macrophage subset.

### VPA suppressed specific cytokine/chemokines in treated operated conjunctiva

**Fig. 3:**
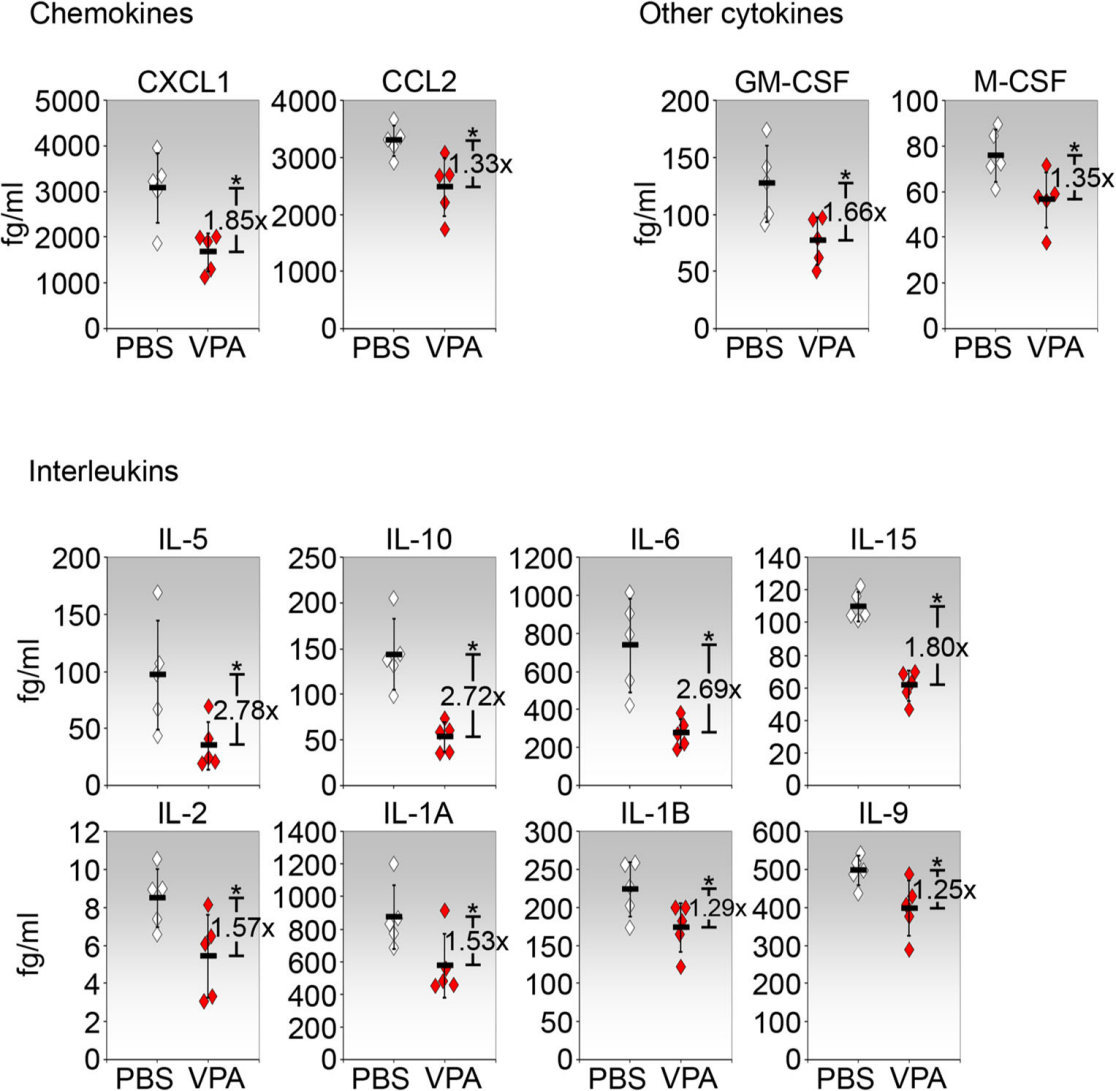
Selective reduction of cytokines/chemokines in the operated conjunctiva treated with VPA. Operated conjunctival tissues were harvested from eyes treated with PBS or VPA for 2 days and subjected to multiplex cytokine assay. Only measurable cytokines and where VPA caused a significant difference relative to PBS controls are shown. Cytokines/chemokines are shown in the order of greatest to lowest in effects exerted by VPA. Values shown are normalized for total protein in each lysate sample. Each sample was pooled from operated conjunctiva of five independent eyes. Five samples were analyzed for each condition/ cytokine (*n* = 5) and the mean concentration ± SD is shown. The fold change due to VPA treatment relative to PBS control is indicated. *p < 0.05

We have previously reported that cytokines/chemokines were elevated in the day 2 operated conjunctiva using multiplexed cytokine analysis [16]. We therefore examined whether VPA has the capacity to alter these cytokine/ chemokine levels. Of the 10 chemokines examined, only CXCL1 and CCL2 were significantly inhibited by VPA (Fig. 3). CXCL2, CXCL5, CXCL9, CXCL10, CCL3, CCL4, CCL5, and CCL11 were not significantly affected by VPA (data not shown). Of the other 7 cytokines/ growth factors evaluated, only GM-CSF and M-CSF were inhibited by VPA (Fig. 3). G-CSF, LIF, TNF-α, and VEGFA were not significantly altered while IFN-ɣ was too low to be detected (data not shown). Of the 15 interleukins interrogated, 8 were significantly suppressed by VPA (Fig. 3). Notably, VPA reduced IL-5, IL-6, and IL-10 by nearly 3-fold (Fig. 3). IL-4 was not significantly altered while the levels of IL-3, IL-7, IL-12p40, IL-12p70, IL-13, and IL- 17 were too low to be detected accurately (data not shown). Hence, VPA modulates specific cytokines to different extents in the operated conjunctiva.

### VPA inhibited specific NF-kB expression in treated operated conjunctiva

**Fig. 4:**
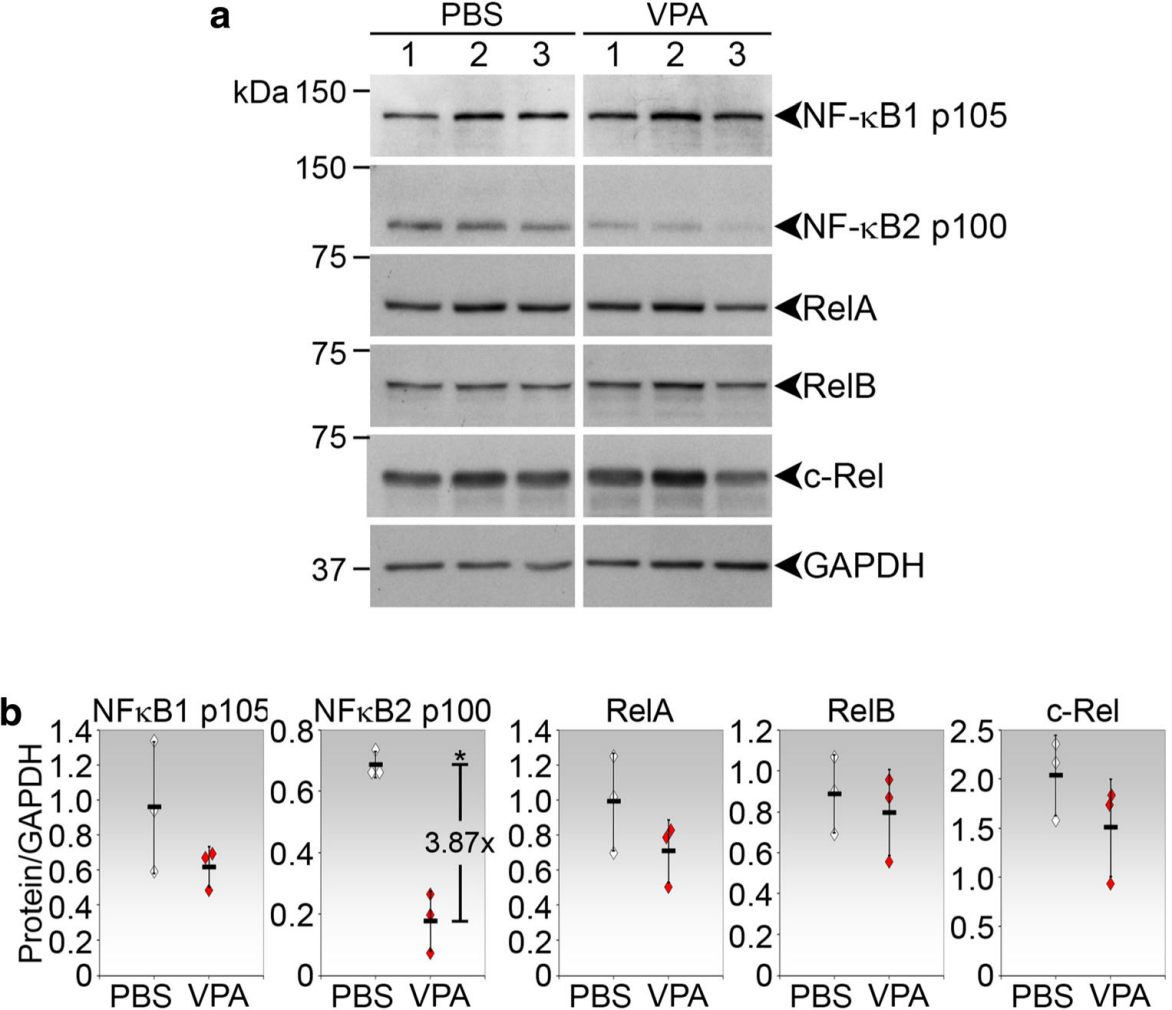
Inhibition of specific NF-қB expression in the operated conjunctiva treated with VPA. Operated conjunctival tissues were harvested from eyes treated with PBS or VPA for 2 days and subjected to immunoblot assay for NF-қB proteins. **a** Immunoblots of three PBS- or VPA-treated samples were probed for the indicated proteins (*n* = 3). Each sample was pooled from the operated conjunctiva of five independent eyes. GAPDH was used to indicate protein loading. **b** Densitometric analyses of NF-қB expression. Densitometric values were normalized against corresponding GAPDH and presented as ratios of the NF-қB and GAPDH units. The mean densitometric ratio ± SD is shown. Where significant, the fold reduction in mean NF-қB expression in VPA compared to PBS treatment is shown. *p < 0.05

The nuclear factor NF-κB pathway is a prototypical proinflammatory signaling pathway that serves as a pivotal mediator of inflammatory responses [20]. To determine the involvement of NF-қB in mediating the anti-inflammatory effects of VPA, we examined the tissue expression of the five NF-қB members: NF-қB1 p105, NF-қB2 p100, RelA, RelB, and c-Rel (Fig. 4a). VPA treatment significantly reduced NF-қB2 p100 by mean 3.87-fold compared to PBS control (Fig. 4b). None of the other NF-қB proteins were significantly affected by VPA treatment. Hence, VPA appears to have a specific inhibitory effect on NF-қB protein expression in the operated conjunctiva.

### VPA suppressed specific cytokine/chemokines in treated conjunctival fibroblasts

**Fig. 5:**
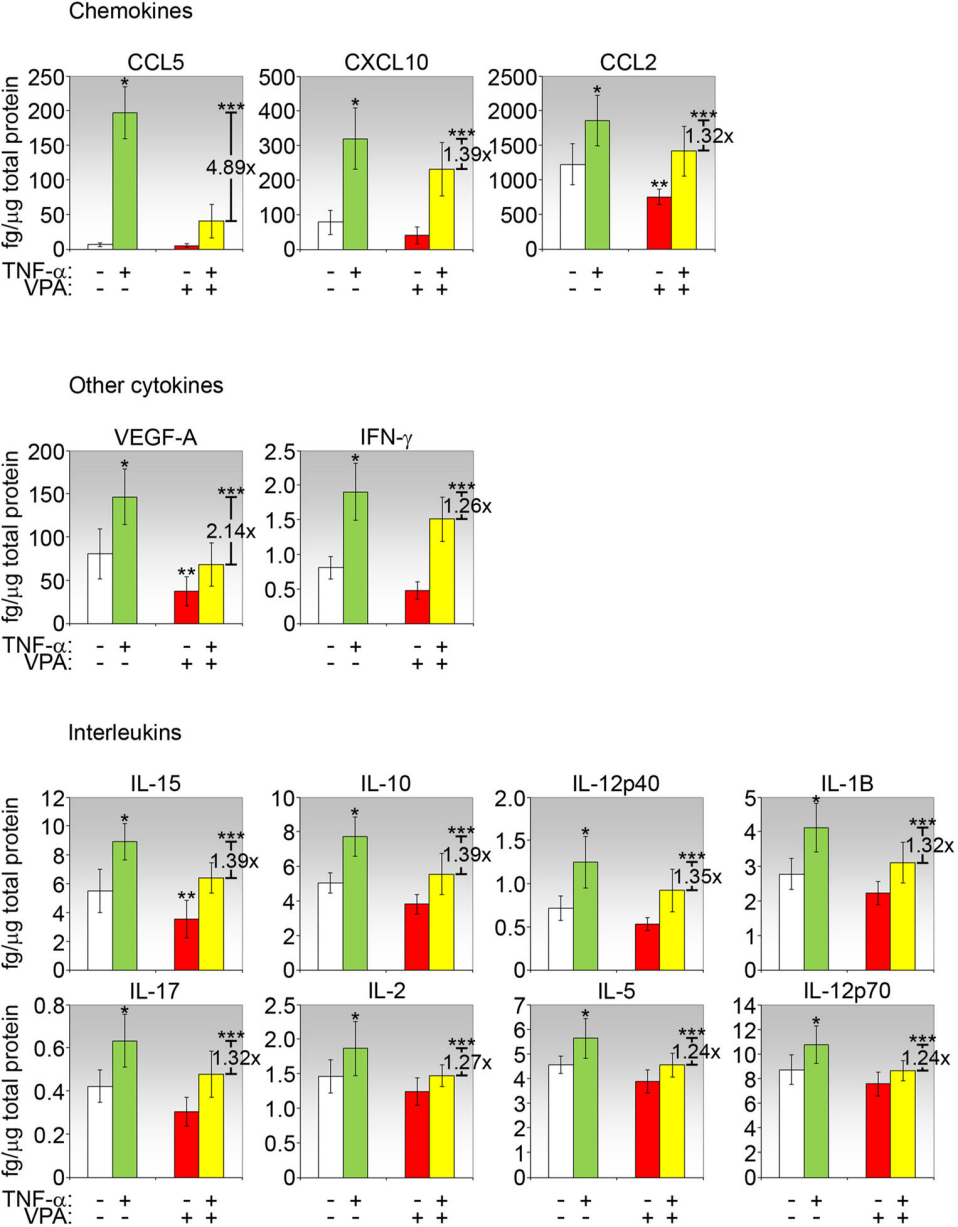
Cytokines/chemokines secreted by conjunctival fibroblasts treated with VPA and/or TNF-α. Culture media from conjunctival fibroblasts treated with PBS or VPA for 2 days were subjected to multiplex cytokine assay. Only measurable cytokines and where VPA caused a significant difference relative to PBS are shown. Cytokines/chemokines are shown in the order of greatest to lowest in effects exerted by VPA. Values shown are the means of three independent experiments, with each experiment performed in triplicate, and calculated as the amount of cytokine/chemokine per μg of total protein in each lysate sample. The fold change due to VPA+TNF-α compared to TNF-α only treatment is indicated where significant. *p < 0.05 comparing TNF-α with no treatment (negative control); **p < 0.05 comparing VPA with negative control; ***p < 0.05 comparing VPA+TNF-α with TNF-α only

We reported previously that conjunctival fibroblasts have the capacity to participate in inflammation by expressing cytokines/chemokines when cultured in a proinflammatory milieu [16]. Moreover, these fibroblasts demonstrated responses to prototypical proinflammatory (TNF-α) or fibrogenic (TGF-β) stimulus that mirror in vivo responses in the inflammatory or fibrotic phase of conjunctival wound healing, respectively [16]. Hence, conjunctival fibroblasts are useful in vitro tools for drug investigation as well as aid understanding of in vivo responses. Evaluation of culture supernatants using the multiplex cytokine assay revealed that VPA significantly suppressed unstimulated fibroblast secretion of CCL2, VEGF-A, and IL-15 (Fig. 5). Moreover, VPA significantly downregulated the induction of selected cytokines/chemokines by TNF-α (Fig. 5). Notably, upregulation of both CCL5 and VEGF-A by TNF-α was significantly reduced by VPA by approximately 5- and 2-fold, respectively. The other cytokines were either not significantly affected by VPA treatment (CCL3, CCL4, CCL11, CXCL9, G-CSF, GM-CSF, M-CSF, LIF, IL-7, IL-9, IL-13) or the values were out of range to be measured accurately under the same experimental conditions (too high: CXCL1, CXCL2, CXCL5, IL-6; too low: IL-3, IL-4). The inhibitory effect of VPA on TNF-α induction of interleukins was also significant, albeit less intense. Since VPA can subdue proinflammatory cytokine secretion by uninduced fibroblasts, this drug may potentially be helpful for preempting inflammation when applied pre-operatively, on top of intra- and post-operative administration.

### VPA inhibited specific NF-kB expression and transcriptional activity in treated conjunctival fibroblasts

**Fig. 6:**
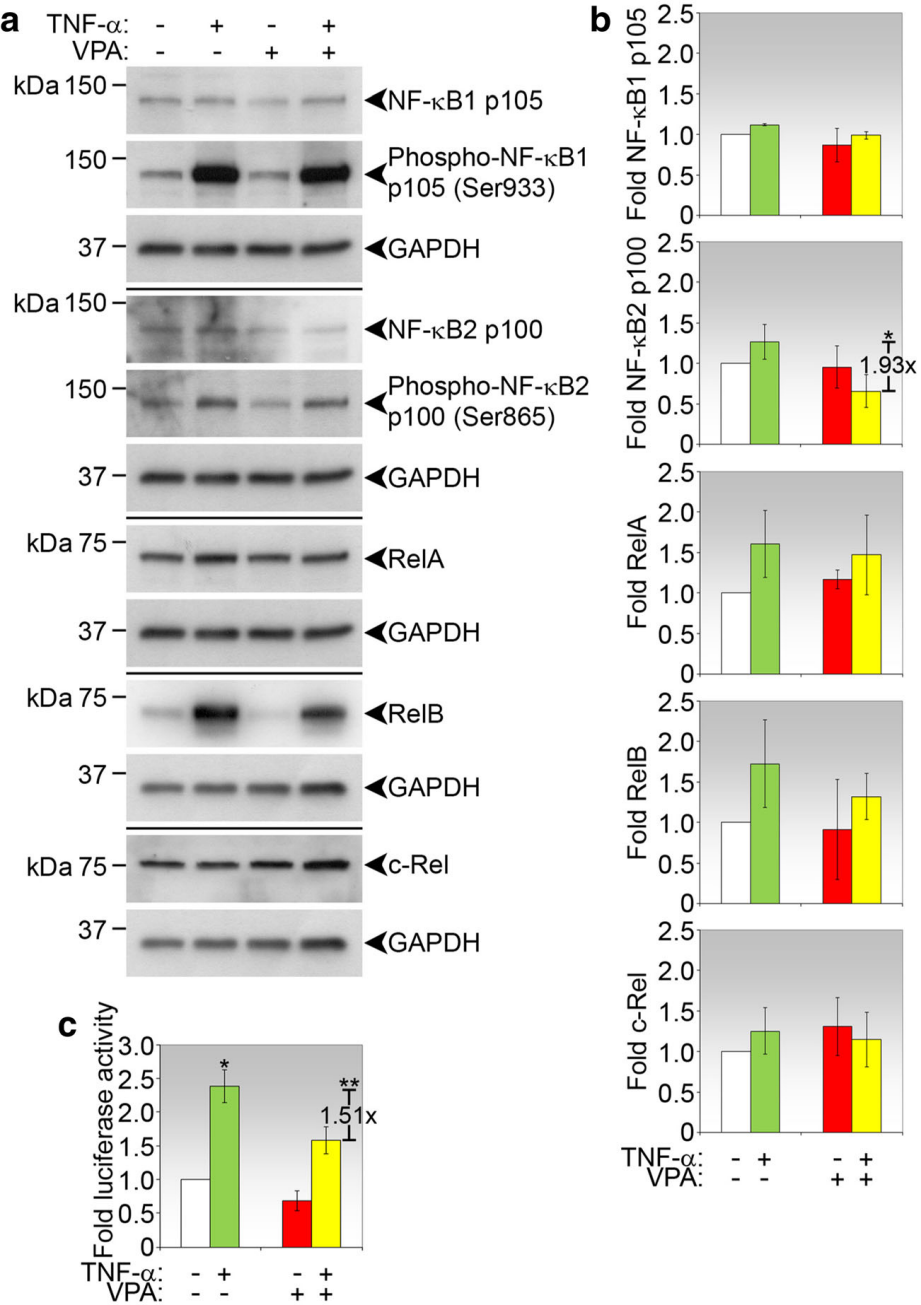
NF-қB expression and activity in conjunctival fibroblasts treated with VPA and/or TNF-α. **a** Representative immunoblots of conjunctival fibroblasts treated as indicated for 2 days and probed for NF-қB proteins. GAPDH for each NF-қB analysis was used to indicate protein loading. **b** Densitometric analyses of NF-қB expression. Densitometric values were normalized against corresponding GAPDH and values shown are calculated as fold changes over no treatment (negative control). The mean fold change ± SD of three independent experiments for each NF-қB and each condition is shown. Where significant, the fold reduction in mean NF-қB expression comparing VPA+TNF-α with TNF-α only is shown. *p < 0.05. **c** NF-қB-dependent transcription reporter assay of conjunctival fibroblasts treated with VPA and/or TNF-α. Fibroblasts were transfected with the NF-қB reporter plasmid and subjected to the indicated treatments for 24 h. Data shown represent fold luciferase activity relative to no treatment (negative control). Values are the means ± SD of three independent sets of experiments. *p < 0.05 comparing TNF-α with no treatment (negative control); **p < 0.05 comparing VPA+TNF-α with TNF-α only

We next examined whether NF-қB expression in conjunctival fibroblasts was also modulated by VPA when stimulated with TNF-α. We first determined that VPA did not inhibit the capacity of TNF-α to phosphorylate NF-қB1 or NF-қB2 (Fig. 6a). However, the presence of VPA in the culture medium containing TNF-α significantly reduced NF-қB2 p100 expression by 1.93-fold (Fig. 6b). The other NF-қB members were not significantly altered by VPA, reiterating this specific effect observed in vivo.

To confirm that VPA interrupts NF-қB-dependent transcription activity, conjunctival fibroblasts were transfected with an NF-қB reporter plasmid. As positive control, TNF-α alone significantly induced NF-қB-dependent promoter activity (Fig. 6c). When co-treated with VPA, the promoter activity was significantly reduced by 1.51-fold (Fig. 6c). Taken together, these data confirm that VPA is effective in inhibiting NF-қB-dependent signaling triggered by TNF-α via modulating NF-қB expression and activity.

## Discussion

Using the mouse model of conjunctival scarring, we have demonstrated that VPA ameliorated the inflammatory phenotype of the operated conjunctiva in a selective and specific manner by modulating both cellular and molecular composition of the post-operative tissue. We further revealed that VPA has the capacity to directly modulate specific cytokines secreted by stimulated conjunctival fibroblasts to corroborate the in vivo findings. These data are important for supporting the application of VPA as anti-inflammatory therapeutic in GFS as well as other inflammatory conditions where specific modulation of inflammatory cell types and/or cytokines may be required for a better clinical outcome.

The dosage of VPA used in the experimental model was previously determined based on effective inhibition of collagen expression in mouse conjunctival fibroblasts while remaining within safe limits, causing less than 10% loss in cell density or viability [13]. We further show here that VPA is likely to be safe for treatment of conjunctival inflammation as it did not induce any overt adverse tissue response, such as visually conspicuous increase in vascularity or inflammation. In fact, VPA-treated eyes appeared similar to PBS-injected ones when imaged. Hence, we propose that VPA, administered in the regimen described here, is a prospective therapeutic for conjunctival inflammation.

The process of tissue repair, beginning with injury, is thought to involve macrophages that assume evolving roles. Indeed, subpopulations of F4/80^+^ macrophages which express variations in marker expression levels, and associated with differential functions during inflammation and tissue repair, are reported in many normal [21] and injured [22] tissues. In this study, we have identified a subset of CD45^high^F4/80^low^ macrophages in the operated conjunctiva which have not been previously described in this tissue. These CD45^high^F4/80^low^ macrophages (CD11b^high^) were specifically modulable by VPA. Similar CD11b^high^F4/80^low^ macrophage subsets have been described in experimental liver injury [23, 24] as well as immune (lupus) [25] and nonimmune kidney disease models (unilateral ureteral obstruction, ischemia, diabetic, rhabdomyolysis-induced acute renal injury) [26–30]. In these models [23, 29], the infiltration of F4/80^low^ subset was dependent on C–C motif chemokine receptor 2 (CCR2) which is the main receptor for CCL2 [31]. Hence, we speculate that the CCL2-CCR2 axis is likely to be involved in the recruitment of CD45^high^F4/80^low^ macrophages in the operated tissue in our mouse model, and CCL2 reduction by VPA may then account for reduction in this macrophage subset. Although CCR2 appeared to preferentially regulate recruitment of CD11B^high^F4/80^low^ subset in liver and kidney studies [23, 29], there may be other explanation for the specificity of VPA effects. There is evidence that in some tissues, a single population of macrophages can be both proinflammatory and pro-repair, and they can switch from one subset to another in response to alteration in micro-environmental factors as tissue repair progresses [32]. On this premise, we postulate that VPA may specifically suppress the conversion of macrophages to the CD45^high^F4/80^low^ subset by modulating the cytokine profile of the inflammatory milieu in the post-operative conjunctiva. Alternatively, by modulating specific transcription activity as an HDAC inhibitor, VPA may directly inhibit the differentiation of macrophages to the CD45^high^F4/80^low^ subset. Whatever the mechanism, it is remarkable that VPA has the capacity to cause a decrease, albeit small, in a macrophage subset.

The implication for the specific modulation of CD45^high^F4/80^low^ macrophages in the inflamed operated tissue is less clear. Since macrophage subsets are implicated in different stages of tissue repair [22], specific reduction of CD45^high^F4/80^low^ (CD11b^high^) macrophages by VPA may refine the therapeutic outcome. In a mouse model of rhabdomyolysis-induced acute kidney injury, CD11b^high^F4/80^low^ macrophages expressed more transcripts of genes involved in disease progression, including *Ccl2*, fibronectin and collagen [30]. CD11b^high^F4/80^low^ depletion via liposomal clodronate in this model resulted in decreased chemokine expression, reduced fibrosis and improved kidney repair and animal survival [30]. In a genetic model of diabetes, reduced CD11b^high^F4/80^low^ macrophage recruitment via CCR2 antagonist was associated with protection from kidney damage [29]. On the other hand, in a mouse model of acetaminophen-induced liver injury, CD11b^high^F4/80^low^ macrophages had an anti-inflammatory expression profile and promoted tissue repair by reducing inflammation via phagocytosis and induction of neutrophil apoptosis [23]. The apparent contradictory roles for CD11b^high^F4/80^low^ macrophages suggest that this macrophage subset may acquire functional properties that are specific for the tissue it infiltrates and/or the disease etiology. The role of CD11b^high^F4/80^low^ macrophages in conjunctival inflammation and tissue repair therefore requires further work to fully understand the impact of VPA on conjunctival inflammation and fibrosis. Nonetheless, our data suggest that VPA is a potential drug for therapeutic targeting of macrophages.

The capacity of VPA to downregulate selective proinflammatory cytokines is important for inhibiting inflammation. This is a key property for therapeutic intervention in surgery, especially since cytokines are critical mediators of the inflammatory process and some of these molecules, including TNF-α, IL-6, and IL-1β, have been described in the context of surgical injury and pain [33, 34]. Given that CD11b^high^F4/80^low^ macrophages may express proinflammatory cytokines including TNF-α, IL-6, IL-1α, and IL-12 [28], and *Ccl2*, *Il1b* and *Il12p40* [30], the reduction of these cytokines in the VPA-treated bleb may be partially attributed to specific reduction of CD45^high^F4/80^low^ macrophages. The simultaneous suppression of GM-CSF and M-CSF by VPA is notable. GM-CSF and M-CSF have been shown to generate opposite responses in macrophages. Based mainly on expression of specific markers, macrophages derived from GM-CSF treatment of monocytes are described as “proinflammatory,” while the M-CSF–generated counterparts are “anti-inflammatory” [35]. At sites of inflammation, macrophages will likely be exposed to both CSFs. This is true for the normal, untreated, operated conjunctiva which contained elevated levels of both GM-CSF and M-CSF levels (data not shown). Based on simple understanding of GM-CSF and M-CSF effects on macrophages, the capacity of VPA to reduce both CSFs, with GM-CSF to a greater extent, may suggest that the balance is in favor of anti-inflammation. However, in vitro experimentation suggests that the inflammation phenotype resulting from combined exposure to both GM-CSF and M-CSF is highly complex [36].

Cytokine downregulation by VPA may also occur by direct modulation of expression, as demonstrated by experimentation on cultured conjunctival fibroblasts. This in vitro profile did not exactly match the observed VPA-treated in vivo cytokine profile since the cellular composition of the operated conjunctiva is infinitely more complex. Moreover, fibroblasts in the tissue will be exposed to many other potent proinflammatory stimuli in addition to TNF-α. Nonetheless, the selectivity of cytokine downregulation by VPA is clear. Common between both in vivo and in vitro profiles is the specific modulation of CCL2 and several members of the interleukin family. Elevated CCL2 is implicated in greater risk of scarring in glaucoma surgery [37, 38]. The capacity of VPA to suppress these cytokines, particularly CCL2 and VEGF-A, by steady-state conjunctival fibroblasts, suggests that pre-treatment with VPA may be advantageous for preempting the full cascade of the inflammatory and angiogenic responses in the aftermath of surgery.

Direct regulation of cytokine production by VPA may occur at the level of gene expression or mechanisms involved in their secretion from cells. Although VPA can potentially regulate protein secretion [39–41], it is unlikely that perturbation of the generic secretory pathway can account for the selective effects observed. We speculate that selectivity may be generated at the gene expression level for specific cytokines which may then in turn influence the production of others. This notion is supported by the specific suppression of NF-қB2 p100 expression both in vivo and in vitro, without affecting its capacity to be phosphorylated by TNF-α. Although VPA appears to specifically alter NF-қB2 p100 expression, this was sufficient to cause a significant reduction in NF-қB-transduced transcription activity provoked by TNF-α. We do not discount the possibility that VPA may modulate other aspects of NF-қB properties. For instance, VPA has been documented to influence NF-қB properties that span regulation of expression to post-translational nuclear translocation [42–49]. Nonetheless, on the level of protein expression, VPA effects on the NF-қB family are highly specific. It is highly likely that the impact of VPA on NF-кB signaling may account for the observed alteration in cytokine production by conjunctival fibroblasts, and the effect may extend to macrophages in vivo [50]. Nonetheless, the implications of VPA selectivity on NF-қB2 p100 in inflammation are not yet clear.

Understanding how the inflammatory response is modulated by therapy is useful for guiding its application during pre-, intra-, and post-operative management of patients. Taken together, our data suggest that VPA will be beneficial as pre-operative and intra-operative therapy to reduce conjunctival inflammation. Since VPA appears to be relatively safe and also has the capacity to reduce collagen production [13], the potential use of this drug in post-operative management of inflammation and fibrosis should be considered.
